# Proprioception deficiency in articular cartilage lesions of the knee

**DOI:** 10.1186/s43019-020-00042-7

**Published:** 2020-05-27

**Authors:** Oday Al-Dadah, Lee Shepstone, Simon T. Donell

**Affiliations:** 1 Translational and Clinical Research Institute, Newcastle University, Framlington Place, Newcastle-upon-Tyne, NE2 4HH UK; 2 Department of Trauma and Orthopaedic Surgery, South Tyneside Hospital, Harton Lane, South Tyneside, NE34 0PL UK; 3 Norwich Medical School, University of East Anglia, Earlham Road, Norwich, NR4 7TJ UK

**Keywords:** Articular cartilage, Patient reported outcome measures, Proprioception, Stabilometry, Mechanoreceptors

## Abstract

**Purpose:**

The purpose of this study is to investigate the proprioceptive function of patients with isolated articular cartilage lesions of the knee as compared to normal controls.

**Methods:**

The Cartilage group consisted of eight subjects with radiologically and arthroscopically confirmed, isolated, unilateral, articular cartilage lesions of the knee (Outerbridge grade III or IV). They were compared to 50 normal controls. Knee proprioception was assessed by dynamic postural stabilometry using the Biodex Balance SD System. Patient-reported outcome measures (PROMs) were used to evaluate all subjects.

**Results:**

Proprioception of the injured knee of the Cartilage group was significantly poorer compared to that of the control group (*p* < 0.001). A significant proprioceptive deficit also was observed when the uninjured knees of the Cartilage group were compared to those in the Control group (*p* = 0.003). There was no significant proprioceptive difference between the injured and the contra-lateral uninjured knee of the Cartilage group (*p* = 0.116). A significant correlation was found between the proprioception measurements of the injured and uninjured knee of the Cartilage group (*r* = 0.76, *p* = 0.030). A significant difference was observed in all PROMs (*p* < 0.001) between the Cartilage and Control groups.

**Conclusions:**

Patients with isolated articular cartilage lesions of the knee had a significant proprioceptive deficit as compared to normal controls. The deficiency was profound and even affected the proprioceptive function of the contra-lateral uninjured knee. This study has shown that articular cartilage lesions have a major influence on knee proprioception. However, it remains uncertain as to whether a proprioceptive deficit leads to osteoarthritis or is a consequence of it.

## Background

Articular cartilage is generally considered an avascular, aneural and alymphatic structure. Consequently, little is known about the role that articular cartilage has in the proprioceptive feedback mechanism of the knee. However, numerous clinical studies have been published regarding the role of the anterior cruciate ligament [1–5] (ACL) and the meniscus [6–8] in knee proprioception. Beard et al. [2] defined proprioception according to three components: joint position sense (JPS) (the static awareness of the position of the joint in space), kinaesthesia (the detection of joint movement and acceleration) and the efferent closed-loop reflex (the reflex response activity which regulates muscle stiffness). The central nervous system receives a collective neural input from peripheral receptors (mechanoreceptors) found within joints, ligaments, tendons, muscles and skin [3, 5, 9, 10]. These include Pacinian corpuscles, Ruffini endings and Golgi tendon organs [5, 11, 12]. Mechanoreceptors are activated by mechanical deformations and send frequency-modulated neural signals to the central nervous system (CNS) that allow for a conscious appreciation of the position of the limb in space [13, 14].

Roberts et al. [15] found poorer proprioception in ACL-deficient patients with associated lateral compartment cartilage lesions as compared to those without cartilage lesions. However, they were unable to ascertain whether the proprioceptive deficit in patients with a cartilage injury was simply the result of a higher energy injury, with more widespread effects overall, or was due to damage of neural structures in the chondral, subchondral and osseous tissue.

We conducted a prospective study assessing knee proprioception using stabilometry. The aim of the study was to investigate the proprioceptive function of patients with isolated articular cartilage lesions of the knee as compared to normal controls.

## Methods

### Subjects

Eight subjects were prospectively recruited to the Cartilage group. Table 1 shows their demographic details. The modified Outerbridge Scale (Table 2) was used to classify the articular cartilage lesions identified [16–20]. These eight patients were found to have grade III or IV articular cartilage lesions in either the medial or lateral tibio-femoral compartments or the patello-femoral compartment. An isolated articular cartilage lesion of the knee was diagnosed by clinical history (i.e., localised pain with or without recent trauma) and examination (i.e., focal tenderness but with normal ligaments and meniscus provocation tests) and MRI scan of the injured knee for all patients in the Cartilage group. The diagnosis was confirmed at the time of knee arthroscopy. The patients in the Cartilage group had a normal contra-lateral knee confirmed by clinical history and examination.

**Table 1:** Demographics of subjects

	Cartilage group (*n* = 8)	Control group (*n* = 50)
Mean age (years) (SD)	34 (9)	25 (5)
Male:female	6:2	35:15
Injured knee (right: left)	6:2	–
Mean height (m) (SD)	1.78 (0.1)	1.75 (0.1)
Mean weight (kg) (SD)	89.8 (31.4)	76.1 (14.4)
Mean BMI (kg/m^2^) (SD)	27.8 (7.1)	24.6 (3.4)

*BMI* body mass index, *SD* standard deviation

**Table 2:** Modified outerbridge classification of articular cartilage lesions [16–20]

Grade	Description of lesion
0	Normal
I	Softening
II	Superficial fibrillation
III	Partial thickness loss of cartilage
IV	Full thickness loss of cartilage (exposed subchondral bone)

All subjects in the Control group (Table 1) had normal knees confirmed by clinical history and examination of both knees and an MRI scan of one knee. Of the 50 subjects who volunteered to join the Control group, 25 underwent an MRI scan of their right knee and the other 25 had an MRI scan of their left knee, alternating side in order of presentation. The Control group data was also used as the normal controls in another published study [8].

Subjects who were 16 to 45 years of age were included. Participants were excluded from the study if there was a concomitant cruciate or collateral ligament tear or meniscal tear of the knee, significant history of ankle or hip pathology, lumbar spine symptoms (including radiculopathy in either limb), neurological or vestibular disease, diabetes or regular use of opiate analgesics was present. In addition, subjects were excluded from the Control group if there was a significant history of any knee pathology was present.

Full approval was received for this prospective study from the Research Ethics Committee and the Research Governance Committee. All subjects signed informed consent forms to participate. This study formed part of the first author's Doctorate thesis. 

### Patient-reported outcome measures

Patient-reported outcome measures (PROMs) where obtained for all subjects in both groups. These included the International Knee Documentation Committee (IKDC) Subjective Knee Score [21, 22] and the Knee Injury and Osteoarthritis Outcome Score [23, 24] (KOOS).

### Proprioception

**Fig. 1:**
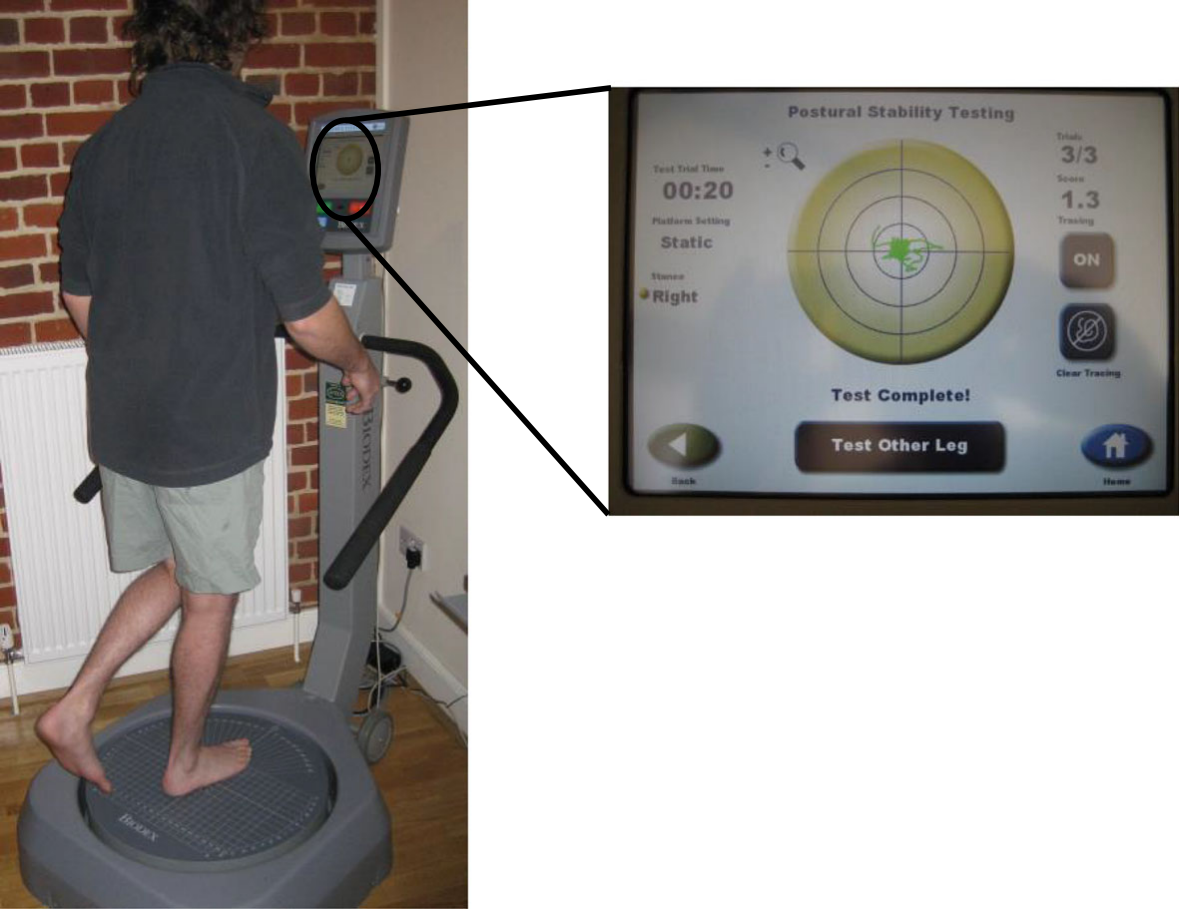
Proprioception assessment using the Biodex Balance SD System. Single-leg stance dynamic postural stabilometry

Proprioception was evaluated using the Biodex Balance SD System (Biodex Medical Systems Incorporated, Shirley, New York) (Fig. 1), which quantitatively measures stabilometry. It has been validated for its use in assessing dynamic single-leg postural stability [25–27]. Dynamic stabilometry is a validated method of measuring knee proprioception [28, 29]. The Biodex Balance SD System consists of a multiaxial moveable platform which detects tilt from 0° to 20° in any direction. In turn, this platform computes an output in the form of an overall stability index (OSI). A low score indicates that the subject has good postural stability (and therefore good proprioception), and a high score reflects poorer stability and proprioception. All subjects in both groups had each leg assessed in bare feet three times for a duration of 20 sec for each test period. The computer output for each leg was calculated from the average of the three tests. The mean OSI result was used as the quantitative measure of proprioception for the purpose of the statistical analyses.

### Statistical analysis

All data variables were assessed for normality of distribution using plotted histograms and the Shapiro-Wilks test. The PROMs data variables for the Cartilage group displayed a normal distribution, but that of the Control group was negatively skewed. The proprioception OSI measurements also displayed a negatively skewed distribution for both groups. Data transformation was implemented for the OSI variables using the natural logarithm, and thereafter, the Log (OSI) data demonstrated a normal distribution for both groups and was used for the purposes of statistical calculations. The results were evaluated using the Mann-Whitney U-test and the independent-sample Student’s *t*-test for between-group analyses as appropriate, paired Student’s *t*-test for within-group analyses and the Pearson product moment test was used for the correlation analysis. The level of statistical significance was set at *p* < 0.05. Statistical analysis was performed using SPSS for Windows version 25.0 (SPSS Inc., Chicago, Illinois).

## Results

Table 3 shows the results of the PROMs analyses. A significant difference was observed between all the PROMs data between the two groups. Table 4 shows the proprioception measurements of both groups, and Table 5 shows the results of their statistical analyses.

**Table 3:** Comparison of patient-reported outcome measures of the Cartilage group (*n* = 8) and Control group (*n* = 50)

	Cartilage Median (IQR)	Control Median (IQR)	*p* value^a^
IKDC Sub.	36 (31–38)	100 (99–100)	< 0.001*
KOOS			
Symptoms	54 (43–54)	100 (96–100)	< 0.001*
Pain	47 (36–53)	100 (100–100)	< 0.001*
ADL	59 (40–63)	100 (100–100)	< 0.001*
Sp. & Rec.	30 (15–45)	100 (100–100)	< 0.001*
QOL	44 (31–44)	100 (100–100)	< 0.001*

*IQR* interquartile range, *IKDC sub* International Knee Documentation Committee Subjective Knee Score, *KOOS* Knee Injury and Osteoarthritis Outcome Score, *ADL* activities of daily living, *Sp. & Rec* sport and recreation, *QOL* quality of life

^a^Mann-Whitney U-test analysis

^*^Statistically significant at < 0.05 level

**Table 4:** Proprioception measurements (Log (OSI))

Group	Mean	(SD)
Cartilage group (*n* = 8)		
Injured knee	1.20	(0.50)
Uninjured knee	0.95	(0.61)
Control group (*n* = 50)		
Right knee	0.49	(0.35)
Left knee	0.52	(0.34)

*SD* standard deviation

**Table 5:** Statistical analysis of proprioception measurements

	Log (OSI)		
	Uninjured knee	Control right	Control left
	*p*-value	*p*-value	*p*-value
	(95% CI)	(95% CI)	(95% CI)
Cartilage group injured knee	0.116	< 0.001*	< 0.001*
	(−0.08-0.59)	(0.43-1.00)	(0.41-0.97)
Cartilage group uninjured knee	–	0.003*	0.004*
	–	(0.17- 0.76)	(0.14-0.73)
Control group right knee	–	–	0.422
	–	–	(−0.04-0.10)

Within group comparison; paired Student’s t-test

Between group comparison; independent-sample Student’s t-test

*Statistically significant at < 0.05 level

A significant difference was observed when the injured knees and the uninjured knees of the Cartilage group were compared to those of the Control group. No significant difference was observed between the injured and uninjured knees of the Cartilage group. Also, no significant difference was observed between the right and left knees of the Control group.

**Fig. 2:**
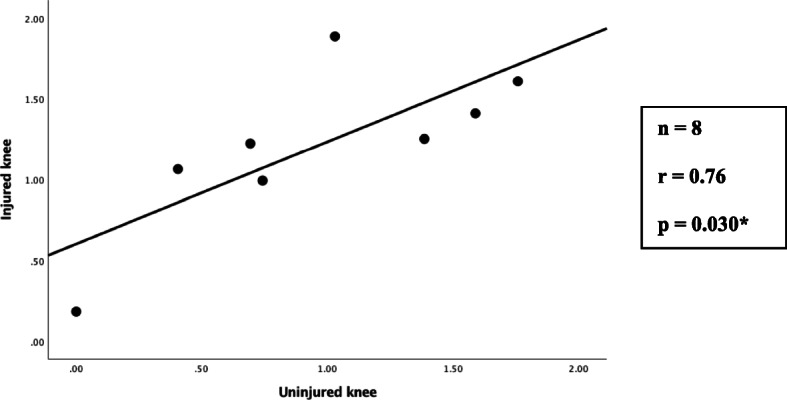
Pearson product moment correlation analysis of proprioception measurements (Log (OSI)) of the injured knee vs. the uninjured knee of the Cartilage group. *Statistically significant at < 0.05 level

Figure 2 illustrates the scatterplot of the Pearson product moment correlation analysis comparing the proprioception measurements of the injured knee to that of the uninjured knee of the Cartilage group. A strong and significant (directly proportional) correlation was found indicating that low Log (OSI) scores in the injured knee were associated with low Log (OSI) scores of the contra-lateral uninjured knee.

## Discussion

The results of this study found that a significant proprioceptive deficit exists as measured by dynamic, single-leg, postural stabilometry in patients with isolated articular cartilage lesions of the knee as compared to normal controls. A significant proprioceptive deficiency was observed in the contra-lateral uninjured knee of the Cartilage group as compared to that of the Control group. We found no significant difference in proprioception between the injured knee and the uninjured knee of the Cartilage group. Furthermore, a strong and significant correlation was found between the latter two variables. The results of the PROMs analysis demonstrated the level of debilitating symptoms endured by patients with articular cartilage lesions of the knee.

The subjects in the Cartilage group had arthroscopically confirmed isolated articular cartilage lesions (Outerbridge grade III or IV) in the presence of an intact ACL and menisci. Both the injured knee and the contra-lateral uninjured knee of the Cartilage group had a significant proprioceptive deficiency. The deficit of proprioceptive function of the uninjured knee was further confirmed by the statistically significant association of the Log (OSI) measurements for both knees of the Cartilage group with a strong correlation coefficient (*r* = 0.76). The latter observation can be substantiated by the suggestion that irregular afferent input from damaged mechanoreceptors in one limb can affect the proprioceptive function of the muscle spindles in the contra-lateral limb [5]. Therefore, the use of the contra-lateral knee is not an appropriate or adequate comparator to serve as a control. A strength of this study was the use of an independent external Control group. This study has shown that articular cartilage lesions have a considerable impact on the proprioceptive function of the knee. Alterations in gait pattern can result from this proprioceptive insufficiency, which in turn can cause unphysiological joint loading and thereby lead to degenerative changes within the joint. The abnormal loading of subchondral bone stimulates nerve fibres. Neural ingrowth into the articular cartilage occurs through the osteochondral junction [30]. The consequence of reduced proprioceptive feedback could also result in repetitive intra-articular trauma leading to progressive cartilage degradation and ultimately osteoarthritis. Poor proprioception leads to poor muscle control [31–33] and can also be a factor in the progression to osteoarthritis.

Various methods for measuring proprioceptive accuracy of the knee have been described in the literature [34]. The different protocols for measuring knee proprioception do not correlate well with each other, and variations in protocol seem to affect measurement outcome. Proprioception may play an important role in knee osteoarthritis; however, this role needs further clarification. A new measurement protocol for knee proprioception needs to be developed. Review articles [34] have advised that future studies need to focus on causes of impaired proprioceptive accuracy in patients with knee osteoarthritis, taking into account that the asymptomatic knee may also have proprioceptive impairments too.

The presence of mechanoreceptors have been demonstrated within articular cartilage [35]. However, as with the meniscus, few clinical studies specifically relate to this topic. In 1991, Barrett et al. [36] first introduced their technique of measuring proprioception using joint position sense (JPS) methods which involved the use of a Thomas splint with a Pearson knee-flexion piece to which a protractor was attached to measure the angle of knee flexion and extension. The principle of this technique formed the basis of knee proprioception assessment in many other studies [3, 6, 37]. This approach has been found to be a reliable and reproducible [36] technique. They investigated knee proprioception in three groups of patients, including subjects with radiologically proven osteoarthritis, patients who had previously undergone a total knee replacement (TKR) and a group of healthy controls. They also assessed the influence of the application of an elastic knee bandage on knee proprioception. Their results showed that the group of patients with osteoarthritis had significantly poorer proprioception as compared to the control group. The patients in the group that had undergone TKR were found to have significantly better proprioception than the osteoarthritic group, but the improvement did not reach the level of proprioceptive acuity of the control group. They found that older subjects had significantly poorer proprioceptive function than younger subjects in the control group. In addition, the elastic bandage was also found to significantly improve proprioception in patients with impaired JPS but had no effect on the control group with normal JPS. The proprioceptive deficit observed in the patients with osteoarthritis may have been due to the loss of cartilage (and therefore joint space narrowing) which resulted in laxity of the joint capsule and ligaments, which in turn influenced mechanoreceptor function. Another probable explanation included the damage incurred to the mechanoreceptors within the joint capsule by the lytic enzymes associated with the osteoarthritic process. Therefore, the proprioceptive changes may have occurred due to a combination of biomechanical and histochemical factors. The restoration of joint alignment and joint space height that accompanied a TKR was also demonstrated to result in significant proprioceptive improvement.

Hunt et al. [38] measured single-leg standing balance using posturography (centre of pressure (COP) movement) obtained from a floor-mounted force platform in individuals with medial compartment knee osteoarthritis. They paradoxically found an inverse relationship between COP movement and radiographic disease severity as well as the number of painful knees. This translated to patients with more severe arthritis or those with bilateral symptoms exhibiting better single-leg standing balance. They justified their unexpected findings by hypothesizing that patients with more advanced arthritis had higher levels of quadriceps and hamstring muscles co-contraction, which would have resulted in stiffer lower extremities and subsequent reductions in COP movement. The results of our present study are consistent with logical findings which may be due to more advanced and reliable equipment used to measure proprioception.

Cho et al. [39] conducted a single-blinded, randomized, controlled clinical trial in female patients with knee osteoarthritis and found that a knee effusion (induced with normal saline intra-articular injection) may also contribute to proprioception deficits in these patients. Van der Esch et al. [40] conducted a cross-sectional study and reported a further proprioception deficit in patients with knee osteoarthritis who have a co-existing medial meniscal tear. We adjusted for these confounders in our study by excluding patients with meniscal tears so as to focus on the sole influence of isolated cartilage lesions.

Possible limitations of the current study include the comparatively higher number of male patients compared to female patients but this was proportionate to the Control group, the slight age difference between the two groups and the number of patients recruited to the Cartilage group; however, the latter was a reflection of the overall low prevalence of patients who fitted the strict inclusion criteria. Nonetheless, the magnitude of the proprioceptive deficit in the Cartilage group was pronounced and convincingly demonstrated strong statistical significance, therefore, spending further time on recruitment was not deemed necessary. The contra-lateral knee of the Cartilage group was thoroughly clinically assessed as being normal. We did not perform an MRI scan or knee arthroscopy of the contra-lateral knee as it was not deemed ethical to subject the patients to further procedures or surgery in the absence of symptoms and a clinical indication.

## Conclusions

In conclusion, these results indicate that a demonstrable proprioceptive deficiency exists in patients with chondral injuries even in the presence of a normal ACL and menisci. As no significant side-to-side differences existed in knee proprioception of the Cartilage group, chondral lesions in one knee can be inferred to impede the afferent neural input and therefore the proprioceptive function of the contra-lateral uninjured knee. This concept was further confirmed by the strong and significant correlation between both knees of the Cartilage group. This study has shown that articular cartilage lesions have a major influence on knee proprioception. However, whether a proprioceptive deficit causes osteoarthritis or it occurs as a consequence of it remains uncertain.

## Data Availability

The datasets developed and/or analysed during the current study are available from the corresponding author on reasonable request.

## References

[1] Barrett DS (1991). Proprioception and function after anterior cruciate reconstruction. J Bone Joint Surg (Br).

[2] Beard DJ, Kyberd PJ, Fergusson CM, Dodd CA (1993). Proprioception after rupture of the anterior cruciate ligament. An objective indication of the need for surgery?. J Bone Joint Surg (Br).

[3] Fremerey RW, Lobenhoffer P, Zeichen J, Skutek M, Bosch U, Tscherne H (2000). Proprioception after rehabilitation and reconstruction in knees with deficiency of the anterior cruciate ligament: a prospective, longitudinal study. J Bone Joint Surg (Br).

[4] Iwasa J, Ochi M, Adachi N, Tobita M, Katsube K, Uchio Y (2000). Proprioceptive improvement in knees with anterior cruciate ligament reconstruction. Clin Orthop Relat Res.

[5] Reider B, Arcand MA, Diehl LH (2003). Proprioception of the knee before and after anterior cruciate ligament reconstruction. Arthroscopy..

[6] Jerosch J, Prymka M, Castro WH (1996). Proprioception of knee joints with a lesion of the medial meniscus. Acta Orthop Belg.

[7] Thijs Y, Witvrouw E, Evens B, Coorevits P, Almqvist F, Verdonk R (2007). A prospective study on knee proprioception after meniscal allograft transplantation. Scand J Med Sci Sports.

[8] Al-Dadah O, Shepstone L, Donell ST (2011). Proprioception following partial meniscectomy in stable knees. Knee Surg Sports Traumatol Arthrosc.

[9] Beynnon BD, Ryder SH, Konradsen L, Johnson RJ, Johnson K, Renstrom PA (1999). The effect of anterior cruciate ligament trauma and bracing on knee proprioception. Am J Sports Med.

[10] Grob KR, Kuster MS, Higgins SA, Lloyd DG, Yata H (2002). Lack of correlation between different measurements of proprioception in the knee. J Bone Joint Surg (Br).

[11] Hogervorst T, Brand RA (1998). Mechanoreceptors in joint function. J Bone Joint Surg Am.

[12] Schultz RA, Miller DC, Kerr CS, Micheli L (1984). Mechanoreceptors in human cruciate ligaments. A histological study. J Bone Joint Surg Am.

[13] Barrack RL, Skinner HB, Buckley SL (1989). Proprioception in the anterior cruciate deficient knee. Am J Sports Med.

[14] Borsa PA, Lephart SM, Irrgang JJ, Safran MR, Fu FH (1997). The effects of joint position and direction of joint motion on proprioceptive sensibility in anterior cruciate ligament-deficient athletes. Am J Sports Med.

[15] Roberts D, Andersson G, Friden T (2004). Knee joint proprioception in ACL-deficient knees is related to cartilage injury, laxity and age: a retrospective study of 54 patients. Acta Orthop Scand.

[16] Cameron ML, Briggs KK, Steadman JR (2003). Reproducibility and reliability of the outerbridge classification for grading chondral lesions of the knee arthroscopically. Am J Sports Med.

[17] Curl WW, Krome J, Gordon ES, Rushing J, Smith BP, Poehling GG (1997). Cartilage injuries: a review of 31,516 knee arthroscopies. Arthroscopy..

[18] Outerbridge RE (1961). The etiology of chondromalacia patellae. J Bone Joint Surg (Br).

[19] Outerbridge RE, Dunlop JA (1975). The problem of chondromalacia patellae. Clin Orthop Relat Res.

[20] Spindler KP, Warren TA, Callison JC, Secic M, Fleisch SB, Wright RW (2005). Clinical outcome at a minimum of five years after reconstruction of the anterior cruciate ligament. J Bone Joint Surg Am.

[21] Irrgang JJ, Anderson AF (2002). Development and validation of health-related quality of life measures for the knee. Clin Orthop Relat Res.

[22] Irrgang JJ, Anderson AF, Boland AL (2001). Development and validation of the international knee documentation committee subjective knee form. Am J Sports Med.

[23] Roos EM, Lohmander LS (2003). The Knee injury and Osteoarthritis Outcome Score (KOOS): from joint injury to osteoarthritis. Health Qual Life Outcomes.

[24] Roos EM, Roos HP, Lohmander LS, Ekdahl C, Beynnon BD (1998). Knee Injury and Osteoarthritis Outcome Score (KOOS)--development of a self-administered outcome measure. J Orthop Sports Phys Ther.

[25] Arnold BL, Schmitz RJ (1998). Examination of balance measures produced by the Biodex Stability System. J Athl Train.

[26] Schmitz R, Arnold B (1998). Intertester and intratester reliability of a dynamic balance protocol using the Biodex Stability System. J Sport Rehabil.

[27] Pincivero DM, Lephart SM, Henry T (1995). Learning effects and reliability of the Biodex Stability System. J Athl Train.

[28] Hewett TE, Paterno MV, Myer GD (2002). Strategies for enhancing proprioception and neuromuscular control of the knee. Clin Orthop Relat Res.

[29] Lephart SM, Pincivero DM, Rozzi SL (1998). Proprioception of the ankle and knee. Sports Med.

[30] Donell S (2019). Subchondral bone remodelling in osteoarthritis. EFORT Open Rev.

[31] Casana J, Calatayud J, Ezzatvar Y, Vinstrup J, Benitez J, Andersen LL (2019). Preoperative high-intensity strength training improves postural control after TKA: randomized-controlled trial. Knee Surg Sports Traumatol Arthrosc.

[32] Mau-Moeller A, Jacksteit R, Jackszis M (2017). Neuromuscular function of the quadriceps muscle during isometric maximal, submaximal and submaximal fatiguing voluntary contractions in knee osteoarthrosis patients. PLoS One.

[33] Petrella M, Gramani-Say K, Serrao PR (2017). Measuring postural control during mini-squat posture in men with early knee osteoarthritis. Hum Mov Sci.

[34] Knoop J, Steultjens MP, van der Leeden M (2011). Proprioception in knee osteoarthritis: a narrative review. Osteoarthr Cartil.

[35] Zimny ML (1988). Mechanoreceptors in articular tissues. Am J Anat.

[36] Barrett DS, Cobb AG, Bentley G (1991). Joint proprioception in normal, osteoarthritic and replaced knees. J Bone Joint Surg (Br).

[37] Jerosch J, Prymka M (1996). Knee joint proprioception in normal volunteers and patients with anterior cruciate ligament tears, taking special account of the effect of a knee bandage. Arch Orthop Trauma Surg.

[38] Hunt MA, McManus FJ, Hinman RS, Bennell KL (2010). Predictors of single-leg standing balance in individuals with medial knee osteoarthritis. Arthritis Care Res.

[39] Cho YR, Hong BY, Lim SH (2011). Effects of joint effusion on proprioception in patients with knee osteoarthritis: a single-blind, randomized controlled clinical trial. Osteoarthr Cartil.

[40] van der Esch M, Knoop J, Hunter DJ (2013). The association between reduced knee joint proprioception and medial meniscal abnormalities using MRI in knee osteoarthritis: results from the Amsterdam osteoarthritis cohort. Osteoarthr Cartil.

